# Effectiveness of minimum retesting intervals in managing repetitive laboratory testing: experience from a Croatian university hospital

**DOI:** 10.11613/BM.2019.030705

**Published:** 2019-10-15

**Authors:** Ivana Lapić, Dunja Rogić, Mirjana Fuček, Ružica Galović

**Affiliations:** Department of Laboratory Diagnostics, University Hospital Center Zagreb, Zagreb, Croatia

**Keywords:** evidence-based practice, clinical laboratory services, pre-analytical phase, cost analysis

## Abstract

**Introduction:**

Inappropriate laboratory retesting can be addressed by implementing minimum retesting intervals (MRI). The aim of our study was to assess the effectiveness of the implemented MRI protocol for inpatients.

**Materials and methods:**

Minimum retesting intervals were applied for 53 laboratory tests. The overall reduction of test requests, reduction in charges and reagent cost savings, frequency of MRI alert appearance as well as the rate of MRI acceptance and ignorance were calculated for a one-year period. Reasons for violating the MRI rule, hospital departments that contributed mostly to MRI rule violation, and the frequency of MRI violations between routine and emergency laboratory were evaluated.

**Results:**

During the one-year period, 106,780 requests violated the MRI rule, which corresponds to 14.8% of all requests received. 13,843 requests were cancelled, yielding a 1.9% reduction of requested tests. High-volume tests, namely complete blood count, C-reactive protein, alanine aminotransferase, gamma-glutamyltransferase and total bilirubin, accounted for 65% of all generated alerts and had the highest alert ignorance (>85%). The highest cancellation rate was observed for tumor markers and autoimmunity tests, for most being at least 50%. Annual charge reduction was 62,641 EUR while reagent cost savings were 11,408 EUR. Tests performed in the emergency laboratory had a higher alert appearance than the same routine tests. The most common reason for MRI violation was clinical justification based on the patient’s condition. Most frequently ignored MRI alerts were in the intensive care unit.

**Conclusion:**

MRI implementation showed limited effectiveness in reducing testing repetition and achieving financial savings, yet provided the basis for future improvements.

## Introduction

Appropriate use of laboratory testing represents an ongoing challenge and is nowadays addressed as an important preanalytical issue that inevitably requires active laboratory participitation and initiative (*1*). The increasing pressure to reduce expenditures in healthcare with an ongoing expansion in the number and availability of laboratory tests has encouraged laboratory professionals to introduce interventions aimed to optimize the use of laboratory testing. These comprise educational strategies, such as informative lectures and dissemination of existing guidelines, as well as various administrative approaches (*2*). The latter usually involve incorporation of software solutions in the laboratory information system (LIS) and/or hospital information system (HIS). Those might include testing algorithms and reflex testing protocols, redesign of the order entry form, adding information on test costs on the request interface or interventions that limit repetitive testing (*2*-*6*). Implementation of interventions at the point of request is a desirable way to guide laboratory utilization because in that way superfluous testing is avoided prior to sample collection, thus both not compromising patient safety and preventing unnecessary costs in terms of blood collection and subsequent medical waste (*7*).

Inappropriate retesting is considered a cause of laboratory overutilization that can be managed through implementation of minimum retesting intervals (MRI) within the laboratory order entry system (*8*). In 2013, the Association for Clinical Biochemistry and Laboratory Medicine (ACB), supported by the Royal College of Pathologists, published recommendations intended to provide assistance for use of MRI. They defined optimal MRI for a large set of laboratory tests based on evidence-based guidelines and best state-of-the-art practice, as well as prerequisites for MRI implementation (*9*). However, there is no universal formula for successful implementation of MRI.

This challenging task can be managed through proper intervention design as well as selection of appropriate tests and MRI, taking into consideration the requirements of the respective setting and possibilities of the used ordering system (*9*). Pop-up alerts are a common way of providing notification and feedback to requesting physicians about inappropriate retesting (*9*, *10*). The so far described approaches differ by the level of allowing to override the MRI rule. Soft-stop approaches imply only small additional effort by the requestor, *i.e*. providing an explanation in a free text field or reasoning through a multiple-choice questionnaire. On the other hand, the hard-stop usually demands a phone call to the laboratory, making it more demanding and occasionally tedious both for the requestor and laboratory staff (*10*, *11*). Therefore, automated functionalities are considered a better option in spite of less satisfactory outcome results (*11*).

Published data shows that the outcomes of MRI interventions can be highly heterogeneous and occasionally even unsatisfactory (*3*, *6*, *12*). It is recommended that any implemented utilization strategy should be monitored, revised and updated on a regular basis (*6*, *13*). Test reduction and cost savings are easily quantifiable measures and usually primary assessed outcomes. However, in an effort to further improve outcome results and identify weak points, a more comprehensive view of the implemented intervention is needed.

In this study, we aimed to assess the functionality and effectiveness of the implemented soft-stop MRI protocol for a large panel of routine and specialized biochemistry, hematology and coagulation tests within the hospital laboratory order entry system designed to reduce duplicate laboratory testing for inpatients. Additionally, underlying reasons for overruling MRI were identified and frequencies of MRI rule violation between requesting wards as well as laboratory settings (urgency *vs*. routine) were investigated.

## Materials and methods

### Setting

University Hospital Center Zagreb is the largest tertiary academic hospital in Croatia that serves in- and outpatients. The Department of Laboratory Diagnostics provides laboratory services in all fields of laboratory diagnostics including emergency, routine and special biochemistry, haematology and coagulation testing, pharmacology and toxicology, diagnostics of inborn errors of metabolism, cytogenetics and molecular diagnostics. The laboratory performs approximately four million tests *per* year, which corresponds to 1000-1200 patients *per* working day. About 60% of all tests are carried out for inpatients.

Laboratory tests are ordered by physicians *via* an electronic laboratory order entry system within the HIS (BIS, IN2 Group, Zagreb, Croatia) that communicates in a two-directional way with the LIS (BioNET LIS, IN2 Group, Zagreb, Croatia).

### Implementation of MRI

Minimum retesting intervals were introduced for a broad range of biochemistry, haematology and coagulation tests in the laboratory order entry system within the HIS. They were restricted to inpatients, referring to the routine and emergency laboratory and were implemented within the whole institution with the exception of the Department of Paediatrics due to the most vulnerable patient population, as well as the Emergency Department due to the urgency of the tests required.

The selection of common biochemistry, haematology and coagulation tests was based on the availability of recommended MRI from the National Minimum Retesting Intervals in Pathology document and expressed clinical demands (*9*). For the autoimmunity test panel, MRI were defined according to the recommendations published by Maher (*14*). However, MRI were further customized to meet the specific needs and requirements posed by clinicians and also based on the frequency at which laboratory tests are requested as part of established diagnostic and treatment protocols at our institution. A unique MRI was introduced *per* each laboratory test. All tests included, together with their respective MRI applied are presented in Table 1.

**Table 1:** Tests included in the study with respective MRI

**Test**	**MRI (days)**	**Test**	**MRI (days)**
**Biochemistry**		**Haematology and coagulation**	
AFP	20	aPTT	1
ALT	2	Complete blood count	1
AST	2	Fibrinogen	1
CA 125	30	PT	1
CA 15-3	30	**Autoimmunity**	
CA 19-9	30	aCL	42
CEA	30	AMA	90
Chromogranin A	30	ANA screening	90
Copper	14	ANCA screening	90
CRP	1	Anti-beta2 GPI	42
CYFRA 21-1	30	anti-CCP	180
Direct bilirubin	2	anti-dsDNA	90
Ferritin	30	anti-histones	90
Folic acid	60	anti-MPO	90
GGT	2	anti-PR3	90
HDL-cholesterol	7	Anti-tTg	90
Hemoglobin A1c	60	ASMA	90
IgG, IgA, IgM	90	C3, C4	14
Iron	30	CH50	30
LDL-cholesterol	7	EMA	90
NSE	30	ENA confirmatory panel (Sm, SS-A, SS-B, Jo-1)	90
NT-proBNP	21	ENA screening	90
PSA	30	Hu, Yo, Ri	90
Total bilirubin	2	Microsomal LKM-1	90
Total cholesterol	7	Rheumatoid factor	180
Triglycerides	7	SLA	90
UIBC	30	/	/
Vitamin B12	60	/	/
MRI - minimum retesting interval. AFP - alpha-fetoprotein. ALT - alanine aminotransferase. AST - aspartate aminotransferase. CA 125 - cancer antigen 125. CA 15-3 - cancer antigen 15-3. CA 19-9 - cancer antigen 19-9. CEA - carcinoembryonic antigen. CRP - C-reactive protein. CYFRA 21-1 - cytokeratin fragment 21-1. GGT - gamma-glutamyltransferase. NSE - neuron specific enolase. NT-proBNP - N-terminal pro brain natriuretic peptide. PSA – prostate specific antigen. UIBC - unsaturated iron binding capacity. aPTT - activated partial thromboplastin time. PT - prothrombin time. aCL - anticardiolipin antibodies. AMA - antimitochondrial antibodies. ANA - antinuclear antibodies. ANCA - anti-neutrophil cytoplasmic antibodies. anti-beta2 GPI - anti-beta2 glycoprotein I antibodies. anti-CCP - antibodies targeting synthetic cyclic citrullinated peptides. anti-dsDNA - anti-double stranded DNA. anti-MPO - anti-myeloperoxidase antibodies. anti-PR3 - anti-proteinase 3 antibodies. anti-tTg - antibodies against tissue transglutaminase. ASMA - anti-smooth muscle antibody. C3, C4 - complement component 3 and 4. CH50 - haemolytic complement activity. EMA - anti-endomysium antibodies. ENA - extractible nuclear antibodies. Sm - Smith antigen. SS-A - Anti Sjögren’s-syndrome-related antigen A. SS-B - Sjögren syndrome type B antigen. Jo-1 - histidyl tRNA synthetase. Mycrosomal LKM-1 - liver-kidney microsomal antibodies. SLA - soluble liver antigen.			

**Figure 1:**
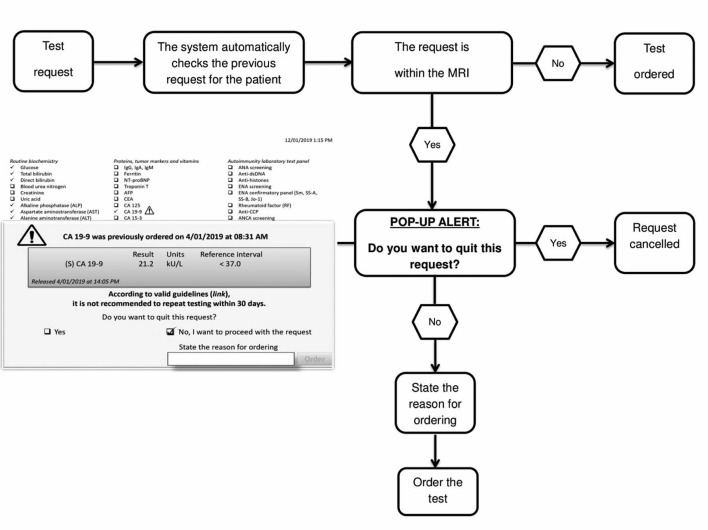
Flow-chart of the MRI functionality with the prototype of the pop-up alert showing a real-life example of an inappropriate re-order of CA 19-9. CA 19-9 - carbohydrate antigen 19-9.

Implementation of MRI required modifications of the existing laboratory order entry system and new functionality was elaborated by information technology (IT) providers. In our system, MRI limits were implemented as follows: if a request is made within the predefined MRI, a pop-up window appears that warns the requestor about the potential requesting inappropriateness according to the defined MRI. The MRI functionality automatically compares each laboratory request made to the previous one for the same patient. The alert is generated for tests requested within the specific MRI, irrespective of whether the result is already available or is still being processed in the laboratory. The pop-up window contains information about the date of the last request, the status of the previous request (as pending or finished with a result attached), defined recommended MRI and the link to the appropriate guideline. At this point, a dual choice is given to the ordering clinician, either to abort the request or continue with it by clicking the appropriate check box. In the latter case, a reason for requesting the test has to be entered in a mandatory field (Figure 1). In this way, the system always enables the clinicians to override the MRI rule if they still consider it clinically appropriate, but at the same time compels them to reconsider the need for the respective laboratory order.

Implementation of MRI required a systematic approach. The intervention had to be thoroughly elaborated prior to introduction, sensibly introduced and continuously monitored to identify areas for improvement. The detailed course of events of MRI implementation is outlined in Table 2.

**Table 2:** Detailed description of the phases of MRI implementation

**Pre-intervention**
Initial idea and design of the most optimal intervention approach based on clinical needs and possibilities of our IT system Presentation of the intervention outline to the institutional Expert Committee and their approval Presentation of the intervention to key senior physicians and agreement on further collaboration Development of a new functionality within the laboratory order entry system Selection of tests being subject to intervention Agreement on the most appropriate MRI
**Intervention**
Implementation of MRI for emergency biochemistry, hematology and coagulation tests at two selected hospital departments (pilot project) Evaluation of results of the one-month pilot project (15) Implementation of MRI for a broad range of tests within the whole institution Education and ongoing help for clinical staff to familiarize with the new functionality
**Post-intervention**
Monitoring of achieved test reductions Calculation of achieved financial savings Focus on special aspects of the intervention (*i.e*. reasons for violating the MRI rule, clinicians and hospital wards with the highest MRI rule violation) Periodical reports about intervention results to the hospital administration and head clinicians Identification of areas for improvement
IT - information technology. MRI - minimum retesting intervals.

### Data collection and analysis

The total number of requests for all laboratory tests included in the MRI project, the number of requests with generated MRI alert and the number of accepted and overruled alerts for each test were collected retrospectively from the HIS for the calendar year 2018. The following data was calculated: overall reduction of test requests, reduction in charges and reagent cost savings, frequency of MRI alert appearance, and the rate of acceptance and ignorance of the MRI rule. The overall reduction of the laboratory tests performed was calculated as the number of alerts accepted that caused laboratory test withdrawal divided by the total number of requests, for each test. To calculate financial savings, we multiplied the number of test withdrawals with the valid national reimbursements fees for each test for the fiscal year 2018, as covered by the Croatian Health Insurance Fund and expressed in euro (EUR). These charges are calculated from all integral costs of the test, including consumable material, reagent, instrument and labour cost, as well as housing and infrastructure, and represent the money provided per test result by the respective national institution for each performed laboratory test. In this context, annulment of laboratory tests contributes to savings for the national healthcare system in general, while calculation of reagent cost savings that arise from a reduction of the tests performed equals to laboratory and/or hospital expense savings. Reagent cost savings were calculated by multiplying the number of cancelled requests with their respective material price.

Frequency of MRI alert appearance was obtained as the number of alerts generated in the total number of requests and presented for test subgroups (*i.e*. biochemistry, haematology, coagulation and autoimmunity). The rate of MRI alert acceptance and ignorance was calculated by dividing the number of accepted and ignored alerts, respectively, with the total number of alerts *per* each test. Hereby we present these rates for high-volume tests as well as selected specialty tests (*i.e*. tumour markers) that were observed to have the highest alert acceptance rate. The reasons for violating the MRI rule were critically reviewed and the clinics whose physicians most often violated the generated MRI alert were identified.

We also assessed the difference of generated and accepted alerts between routine and emergency requests by comparing data for biochemical tests performed in both settings. Data was tested for normality using the Shapiro-Wilk test. For data analysis, comparison of independent proportions was used and Bonferroni correction for multiple testing was applied, P value of 0.006 (0.05/8 = 0.00625) was considered statistically significant. Statistical analysis was performed using MedCalc statistical software, version 14.12.0 (MedCalc, Ostend, Belgium).

## Results

Table 3 summarizes the number of requests for groups of tests being subject to the MRI intervention, the number of generated MRI alerts, test cancellation rates and respective reduction of tests performed, as well as obtained reductions in charges and reagent cost savings. The reduction of charges accounted for 2.3% of total annual charges.

**Table 3:** Number of ordered tests subject to MRI intervention, generated MRI alerts, tests cancelled following the MRI alert, reduction of tests performed, annual reduction in charges and reagent cost savings

**Group**	**Tests ordered,** **N**	**MRI alerts generated,** **N (%)**	**Alerted tests cancelled,** **N (%)**	**Reduction of performed tests (%)**	**Annual reduction in charges (EUR)**	**Annual reagent cost savings (EUR)**
Biochemistry	432,429	58,341 (13.5)	9268 (15.9)	2.1	38,222	6367
Haematology and coagulation	275,329	47,750 (17.3)	4242 (8.9)	1.5	17,606	3121
Autoimmunity	14,421	689 (4.8)	333 (48.3)	2.3	6813	1920
Total	722,179	106,780 (14.8)	13,843 (13.0)	1.9	62,641	11,408
MRI - minimum retesting intervals.						

**Figure 2:**
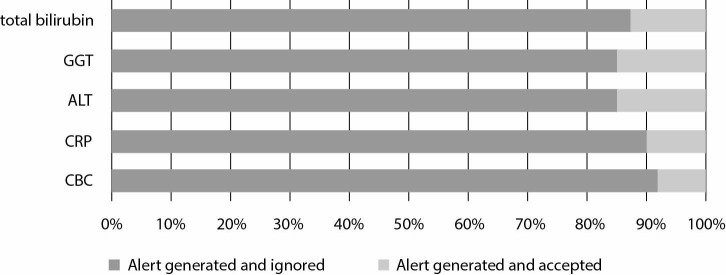
The highest volume tests included in the study with their respective rates of acceptance and ignorance of the MRI rule. MRI - minimum retesting intervals. ALT - alanine aminotransferase. CRP - C-reactive protein. GGT - gamma-glutamyltransferase. CBC - complete blood count.

The highest alert appearance was observed for high-volume tests, namely complete blood count (CBC), followed by C-reactive protein (CRP), alanine aminotransferase (ALT), gamma-glutamyltransferase (GGT) and total bilirubin. These five tests account for 65% of all generated alerts. Interestingly, the observed overall ignorance rates for these frequently ordered tests were among the highest, with over 85% of alert ignorance (Figure 2).

**Figure 3:**
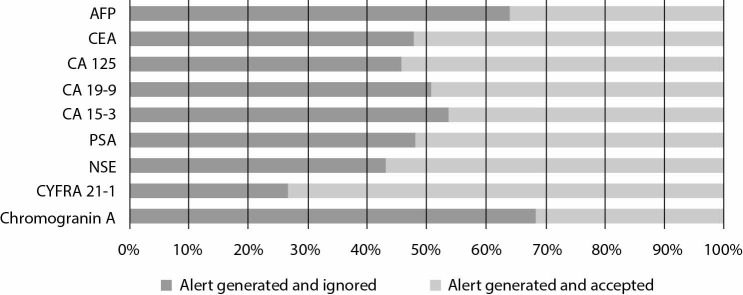
Alert acceptance and ignorance of MRI alerts for tumour markers included in the study. MRI - minimum retesting intervals. AFP - alpha-fetoprotein. CEA - carcinoembryonic antigen. CA 125 - cancer antigen 125. CA 15-3 - cancer antigen 15-3. CA 19-9 - cancer antigen 19-9. PSA - prostate specific antigen. NSE - neuron specific enolase. CYFRA 21-1 - cytokeratin fragment 21-1.

On the other hand, the highest alert acceptance and therefore test cancellation rate was observed for tumor markers (Figure 3). Similarly, high test cancellation following the MRI alert was observed for autoimmunity tests, for most tests being at least 50%, yielding a cumulative cancellation of 48% alerted requests.

**Figure 4:**
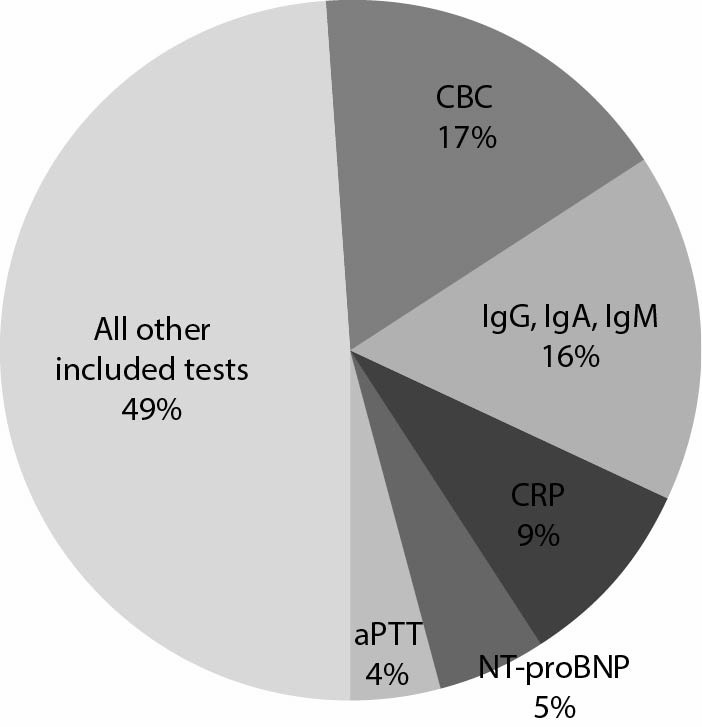
The contribution of single tests to the overall achieved reduction in charges; CBC - complete blood count. IgG, IgA, IgM - immunoglobulins G, A, M. CRP - C-reactive protein. NT-proBNP - N-terminal pro brain natriuretic peptide. aPTT - activated partial thromboplastin time.

**Figure 5:**
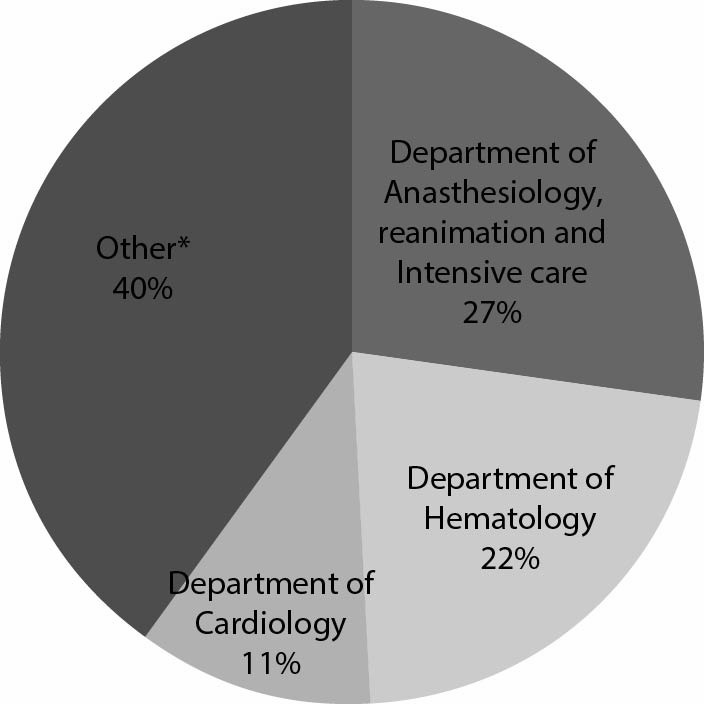
Hospital departments that most frequently violated the MRI rule. MRI - minimum retesting intervals. *Other hospital departments by frequency: Department of Internal Medicine (10%), Department of Oncology (7%), Department of Surgery (6%), Department of Neurology (4%) and with minor contribution Department of Neurosurgery, Department of Pulmology, Department of Ginecology, Department of Urology, Department of Otorhinolaryngology, Department of Rheumatology and Rehabilitation and Department of Psychiatry.

The highest reduction in charges *per* single test was achieved for CBC, followed by immunoglobulins and CRP, as shown in Figure 4. Annulment of CBC requests was the single most prominent contributor to the overall reagent cost reduction with a saving of 2205 EUR (19% of the total amount). Physicians from three hospital departments were identified to contribute to 60% of all violated MRI rules, as shown in Figure 5. Table 4 lists the reported reasons for ordering the requested laboratory test despite the MRI rule. The comparison of the differences of alert appearance and acceptance between biochemical tests performed both in routine and emergency laboratory is presented in Table 5.

**Table 4:** Reasons for violating the MRI rule by frequency

**Reasons for violating the MRI rule, %**	
Physician considered clinically justifiable to order the test based on patient’s condition	40.4
Laboratory monitoring as part of established diagnostic procedures	19.0
Monitoring of critically-ill patients	16.7
Reason for ordering not stated clearly	11.6
Sepsis	6.0
Preanalytical errors (i.e. haemolysis, clot, scarce sample volume, etc.) in the first sample that required repeated sampling	1.9
Therapy monitoring	1.7
Postoperative laboratory testing	1.6
Preoperative laboratory testing	0.9
Other reasons	0.2
The total number of requests with MRI rule violation was 92,937. MRI - minimum retesting intervals.	

**Table 5:** Difference in the frequency of MRI alert appearance and acceptance for biochemical tests performed both in routine and emergency laboratory

**Test**	**Requests with alerts,** **N (%)**			**Requests with accepted alerts,** **N (%)**		
	Routine laboratory	Emergency laboratory	P	Routine laboratory	Emergency laboratory	P
**ALT**	3388 (9.1)	5714 (23.8)	< 0.001	522 (15.4)	839 (14.7)	0.382
**AST**	2599 (10.1)	2498 (18.7)	< 0.001	315 (12.1)	260 (10.4)	0.061
**CRP**	4142 (9.1)	12,426 (25.9)	< 0.001	546 (13.2)	1143 (9.2)	< 0.001
**Direct bilirubin**	837 (6.6)	689 (10.7)	< 0.001	60 (7.2)	52 (7.5)	0.901
**GGT**	3233 (8.9)	4671 (21.5)	< 0.001	489 (15.1)	705 (15.1)	0.975
**NT-proBNP**	313 (9.8)	446 (34.2)	< 0.001	84 (26.8)	109 (24.4)	0.507
**Total Bilirubin**	4102 (11.5)	4519 (22.1)	< 0.001	550 (13.4)	553 (12.2)	0.102
**Total**	18,614 (9.5)	30,963 (22.9)	< 0.001	2566 (13.8)	3661 (11.8)	< 0.001
Bonferroni correction for multiple testing was applied, P < 0.006 was considered statistically significant. ALT - alanine aminotransferase. AST - aspartate aminotransferase. CRP - C-reactive protein. GGT - gamma-glutamyltransferase. NT-proBNP - N-terminal pro brain natriuretic peptide.						

## Discussion

The present study shows that introduction of MRI through a computerized alert system has limited effectiveness in terms of reducing repetitive laboratory testing and achieving financial savings. Furthermore, introduction of MRI provides an insight into the most common underlying causes of MRI violation and addresses the need for introducing MRI tailored to the specific clinical setting. It also highlights the importance of continuous monitoring of MRI intervention outcomes in order to identify possible weaknesses and introduce improvements.

The overall test reduction and annual savings are comparable to the results of a similarly extensive MRI implementation through a hard-stop principle, while other studies yielded better outcome results, regardless of the type of approach (*3*, *11*, *12*, *15*-*17*). The cancellation rate of alerted requests was not as high as in previously reported studies. While the soft-stop similar to ours introduced by Lippi *et al*. resulted in a cancellation of 77% alerted requests, Procop *et al.* evidenced that application of a soft-stop yielded a reduction of 43.6% duplicate orders (*11*, *12*). However, hard-stop principles were shown to be more effective, yet bearing a huge disadvantage of not being automated (*11*, *17*). This variety of results clearly indicates that outcomes from MRI interventions cannot be generalized and that they largely depend on the intervention design, tests included, MRI applied and specific requirements of each setting.

Our study identified high-volume tests as a convenient target for MRI intervention, a fact that was already proven in earlier studies (*16*, *18*, *19*). Despite consistently low annulment rates as well as low unit price, their numerosity contributed substantially to the savings achieved. The very high rate of alert ignorance might either indicate that the defined time frame was inappropriate or more probably, underlines the simplicity to overrule the MRI alert through the applied soft approach. On the other hand, the highest alert acceptance and subsequent test cancellation observed for tumour markers and autoimmunity tests provide a rationale to subject these tests to MRI intervention. Both are not recommended as screening tests in the general population, their use is limited to selected clinical indications and repetitive testing is appropriate mainly for disease monitoring (*9*, *14*). Despite that, published data highlights their inappropriate and excessive ordering, that not only causes a significant economic burden but inevitably induces unnecessary second level follow-up investigations that can compromise patient safety and additionally increase costs (*14*, *20*-*22*). Moreover, most autoimmunity tests are time-consuming and at least partly, performed manually. Also, since these tests have longer turnaround times, MRI alert can serve as a useful reminder of the previous request.

Higher MRI alert generation in the emergency compared to the routine laboratory as well as frequent violation of the MRI rule at hospital wards intended for critical care or treatment of serious systemic conditions suggest that the same MRI for a single test should not be universally applied, but rather tailored to the needs of the specific clinical setting. The most commonly submitted reasons for violating the MRI rule also support this issue. In fact, this is also addressed in the valid MRI recommendations (*9*). Analysis of reasons for MRI violation yielded two additional technical drawbacks of the MRI functionality. Firstly, the minimal number of characters is not predefined, thus allowing overruling of MRI by insertion of a single punctuation mark or random letters. Secondly, the MRI alert is triggered in cases when the request is repeated due to a preanalytical error of a previous sample. It is reasonable to assume that these software drawbacks, at least partly, decreased the effectiveness of the MRI intervention. Therefore, we strongly advocate the design of the MRI functionality in a way to overcome these pitfalls.

Our study has some limitations. It is obvious that some technical and logistical improvements of the MRI intervention are needed. Moreover, this study would benefit from analysis of possible adverse consequences and potential delays in testing due to the implemented MRI. However, we have hardly received any complaint throughout the whole period of active use of MRI. We assume it is because our system allows to order the test fairly easily at all times and the intervention was thoroughly discussed with clinical staff prior to introduction. Also, it would be valuable to study the savings achieved from unneeded downstream diagnostic and therapeutic procedures that were initially avoided by the cancellation of the requested laboratory test. Finally, the inclusion of paediatric population could be considered as a further step since they are known to be most susceptible to iatrogenic anemia from too frequent diagnostic blood testing (*23*).

In conclusion, implementation of MRI through the described soft approach showed limited effectiveness in reducing repetitive laboratory testing and providing financial savings. However, we believe that these are promising results of the challenging initiative to optimize laboratory retesting through the application of MRI for a large battery of tests at once. Indeed, in an era of continuous financial cutbacks as well as rising awareness about patient safety, every single possibility that can contribute to both financial savings and sparing patients from unneeded diagnostic procedures is welcome, especially when the applied system is running properly and in an automated manner. The study gives rise to future improvements of the MRI protocol and its conceptual approach can serve as a model for implementing similar intervention in any clinical laboratory setting.
